# Workplace Secondhand Tobacco Smoke Exposure Among U.S. Nonsmoking Workers, 2015

**DOI:** 10.15585/mmwr.mm6827a2

**Published:** 2019-07-12

**Authors:** Chia-ping Su, Girija Syamlal, Sara Tamers, Jia Li, Sara E. Luckhaupt

**Affiliations:** ^1^Epidemic Intelligence Service, CDC; ^2^Division of Field Studies and Engineering, National Institute for Occupational Safety and Health, CDC; ^3^Respiratory Health Division, National Institute for Occupational Safety and Health, CDC; ^4^Division of Science Integration, National Institute for Occupational Safety and Health, CDC.

Secondhand tobacco smoke (SHS) exposure contributes to ill health and disease, including heart disease, lung cancer, and stroke (*1*). Although cigarette smoking has declined among U.S. workers, workplace exposure to SHS remains high, particularly among workers in certain industries, such as construction (*2*,*3*). Implementation of smoke-free laws has proven to be beneficial in reducing SHS exposure in general (*1*). CDC analyzed data from the 2015 National Health Interview Survey (NHIS) Occupational Health Supplement to assess the prevalence of self-reported workplace SHS exposure among nonsmoking workers by smoke-free policy status in the workers’ states of residence and in detailed industry categories and subcategories. In 2015, 19.9% of nonsmoking workers reported any exposure to SHS at work during the 12 months preceding the interview, and 10.1% reported frequent exposure (twice a week or more). Nonsmoking workers who resided in states with comprehensive smoke-free laws in all three categories of venues (private worksites, bars, and restaurants) were least likely to report frequent exposure to workplace SHS. Nonsmoking workers employed in the commercial and industrial machinery and equipment repair and maintenance industry reported the highest prevalences of any workplace SHS exposure (65.1%), whereas the construction industry had the highest reported number of exposed workers (2.9 million); these industry categories/subcategories include outdoor workplaces and other settings that are unlikely to be protected by smoke-free laws. Identifying specific at-risk workplaces and implementing targeted intervention strategies could help reduce SHS exposure at work and protect workers’ health.

NHIS is conducted annually by CDC to produce nationally representative information on the health of the U.S. civilian, noninstitutionalized population, using a multistage clustered sample design. In 2015, CDC’s National Institute for Occupational Safety and Health (NIOSH) sponsored an Occupational Health Supplement to NHIS to collect information on the prevalence of several work-related conditions and exposures in the U.S. working population, including workplace SHS exposure. For this analysis, CDC included adults aged ≥18 years who were employed* during the week before the interview. Assessment of workplace SHS exposure was based on responses to the question “During the past 12 months, while at work, how often were you exposed to tobacco smoke from other people?” The response options were “never”; “less than twice a week”; “twice a week or more, but not every day”; and “every day.” “Any exposure” to SHS was defined as any response other than never. “Frequent exposure” to SHS was defined as twice a week or more. Regarding state smoke-free policies, this report focuses on smoking restrictions in three categories of venues: private worksites, restaurants, and bars, because these venues are major sources of SHS exposure for nonsmoking workers (*4*). The workers’ states of residence were classified, according to the 2015 smoke-free law status in the three categories of venues, into four categories: 1) no law or noncomprehensive law (e.g., law allowing smoking in designated areas or areas with separate ventilation); 2) 100% smoke-free in one venue category; 3) 100% smoke-free in two venue categories; and 4) 100% smoke-free in all three venue categories (comprehensive). These data were obtained from CDC’s State Tobacco Activities Tracking and Evaluation System database.^†^

Free text responses regarding workers’ current industry were coded to U.S. Census 4-digit industry codes by trained coders and recoded into 78 detailed industry recode categories. Exposure prevalence and 95% confidence intervals were calculated for workers in all industry recode categories and for U.S. Census industry codes that were within recode categories with high reported prevalence of SHS exposure (i.e., subcategories) that had adequate sample sizes. The number of exposed workers in each category was calculated by multiplying the prevalence by the weighted estimated population size. All analyses were weighted to be representative of U.S. civilian noninstitutionalized adults. Two-tailed tests of significance (α = 0.05) were performed to compare the percentages of nonsmoking workers in different groups of states or industry categories reporting SHS exposure. For the industry comparisons, the simple recode category “Information Industries,” which had an exposure prevalence similar to that among all workers, was used as the reference group to identify groups with significantly high prevalences. Most variables used for this study are included in the 2015 public-use data sets, but state of residence and U.S. Census 4-digit industry codes are restricted. The restricted variables were accessed through CDC’s National Center for Health Statistics Research Data Center after the study proposal was approved by the Research Data Center. Data analyses were conducted using SAS-Callable SUDAAN (release 11.0.1; RTI International) within SAS (version 9.3; SAS Institute) to account for the complex sample.

In 2015, 19.9% of nonsmoking workers reported any exposure to workplace SHS during the 12 months before the interview; 10.1% reported frequent exposure. Across all industries, workers who resided in states with comprehensive smoke-free laws in all three categories of venues (private worksites, restaurants, and bars) reported significantly lower prevalences of frequent exposure to workplace SHS (8.6%) than did those residing in states with smoking restriction laws in one category of venue (12.2%) or no smoking restriction laws (11.0%) (Figure). None of the differences in any SHS exposure among workers in state smoking restriction categories was significant. Across all states, self-reported workplace SHS exposure varied by detailed industry categories and subcategories, with several industry groups reporting prevalences of exposure higher than that of the reference industry group (Table). Workers in the commercial and industrial machinery and equipment subcategory within the repair and maintenance industries category had the highest reported prevalence of any workplace SHS exposure (65.1%), followed by workers in the other transportation subcategory, which includes air, rail, pipeline, and scenic and sightseeing transportation (55.8%). The construction industry category had the highest number of nonsmoking workers reporting any SHS exposure (2.9 million).

**FIGURE:**
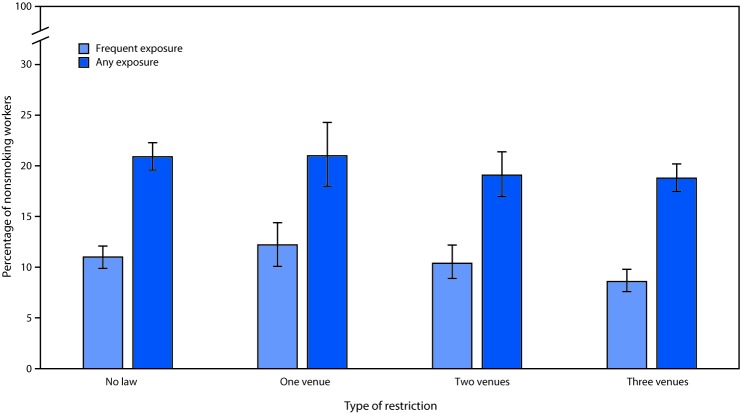
Percentage* of nonsmoking workers reporting any and frequent^†^ workplace exposure to secondhand smoke, by type of restriction^§^^,¶^ of smoke-free indoor air legislation in state of residence — United States, 2015 * With 95% confidence intervals indicated with error bars. ^†^ ≥2 times per week. ^§^ Type of restriction: *No law* = no law or noncomprehensive law (e.g., law allowing smoking in designated areas or areas with separate ventilation) (Alabama, Alaska, California, Connecticut, Georgia, Kentucky, Mississippi, Missouri, Oklahoma, South Carolina, Texas, and Virginia); *One venue* = 100% smoke-free in one venue category (Arkansas, Idaho, New Hampshire, Pennsylvania, and Tennessee); *Two venues* = 100% smoke-free in two venue categories (Florida, Indiana, Louisiana, Nevada, and North Carolina); *Three venues* = 100% smoke-free in three venue categories (Arizona, Colorado, Delaware, District of Columbia, Hawaii, Illinois, Iowa, Kansas, Maine, Maryland, Massachusetts, Michigan, Minnesota, Montana, Nebraska, New Jersey, New Mexico, New York, North Dakota, Ohio, Oregon, Rhode Island, South Dakota, Utah, Vermont, Washington, and Wisconsin). ^¶^ Venue categories include private worksites, restaurants, and bars.

**TABLE:** Percentage of nonsmoking persons exposed to secondhand smoke at work, by industry categories and subcategories* with high prevalence^†^ of any exposure and frequent^§^ exposure to secondhand smoke — United States, 2015

Industry category/Subcategory	Estimated population size (x 1,000)	% Exposed (95% CI)	
		Any exposure	Frequent exposure
**Repair and maintenance**	1,785	45.2 (36.2–54.5)	28.8 (21.5–37.4)
Commercial and industrial machinery and equipment repair and maintenance	279	65.1 (45.1–81.8)	38.4 (20.6–59.9)
Automotive repair and maintenance	1,105	47.3 (35.0–59.9)	29.6 (20.6–40.4)
**Transportation**	3,067	38.8 (33.0–44.8)	25.5 (20.1–31.9)
Other transportation^¶^	218	55.8 (30.2–79.3)	44.5 (21.7–69.8)
Services incidental to transportation	219	43.9 (30.3–58.2)	NS
Taxi and limousine service	232	43.6 (22.8–66.2)	NS
Truck transportation	1,272	41.4 (31.1–52.3)	29.1 (20.1–40.2)
**Forestry and logging**	88	52.9 (24.5–79.5)	NS
**Beverage and tobacco product manufacturing**	169	48.7 (26.6–71.3)	NS
**Museums, historical sites, and similar institutions**	290	47.6 (26.5–69.7)	NS
**Construction**	6,959	41.9 (37.9–46.1)	22.3 (18.9–26.1)
**Accommodation**	1,348	36.6 (27.1–47.2)	28.2 (19.6–38.8)
**Performing arts, spectator sports, and related**	760	36.5 (24.9–49.9)	NS
**Motor vehicle and parts dealers**	1,254	35.6 (25.2–47.6)	NS
**Information (reference group)**	2,995	20.4 (15.2–27.0)	10.3 (6.7–15.5)

**Abbreviations:** CI = confidence interval; NS = not significantly different from reference group.

* Not all subcategories within each category are shown.

^†^ The estimates of prevalence in all categories/subcategories shown were significantly higher than that of the reference group (p<0.05).

^§^ ≥2 times per week.

^¶^ Includes air, rail, pipeline, and scenic and sightseeing transportation.

## Discussion

Nonsmoking workers residing in states with comprehensive smoke-free laws reported significantly lower prevalences of frequent exposure to workplace SHS. Moreover, SHS exposure among nonsmoking workers also significantly varied by industry. During 2013–2014, one in four U.S. nonsmokers reported exposure to SHS (*5*), and an estimated 41,000 deaths among nonsmoking adults were associated with SHS exposure (*1*). Furthermore, workplace SHS exposure has been recognized as one of the top occupational hazards that contributes substantially to the prevalence of occupational cancer among nonsmokers (*6*). During 2000–2015, the number of states with smoke-free laws that prohibited smoking in indoor areas of worksites, restaurants, and bars increased from none to 27 (*4*). In this report, workers residing in states with smoke-free laws in all three venue categories were least likely to report frequent exposure to workplace SHS. Previous studies have revealed that the absence of a policy restricting or prohibiting smoking at the worksite put workers at higher risk for workplace SHS exposure (*7*). Despite the considerable progress in implementation of smoke-free laws over the past 2 decades, this analysis found that even in states with smoke-free laws in three categories of venues, 8.6% of nonsmoking workers reported frequent workplace SHS exposure. This finding suggests that certain workplaces might be outside the scope of most smoke-free laws.

Based on NHIS data for 2014–2016, 34.3% of workers in the construction, 30.4% of workers in the mining, and 30.2% of workers in the transportation industries used some form of tobacco (*8*). Higher smoking prevalences among workers employed in these industries might lead to exposure of their nonsmoking coworkers to SHS. Previous findings of higher tobacco use and SHS exposure among workers in the construction industry are consistent with current findings (*3*,*8*). The industry subcategories with the highest prevalences of reported SHS exposure in this study and the industry category with the highest number of exposed workers (construction) include outdoor workplaces and other settings that are unlikely to be protected by smoke-free laws. A recent study determined that indoor workers who reported working at a worksite having a 100% smoke-free policy had significantly lower odds of smoking combustible tobacco than did those reporting a partial or no smoke-free policy (*9*). Enhanced and sustained efforts to protect nonsmoking workers through comprehensive smoke-free laws and implementation of smoke-free workplace policies by employers can benefit public health.

The findings in this report are subject to at least five limitations. First, all information in NHIS, including work characteristics and SHS exposure, was self-reported at the time of interview and might be subject to reporting bias. Second, although NHIS records state of residence, some workers might work outside the states in which they reside or in multiple states where smoke-free laws might differ. Third, estimates for SHS exposure for some groups were unreliable because of small sample sizes and were therefore suppressed. Small sample sizes within individual industry groups also precluded analyses that combined the state and industry variables. Fourth, the study only accounted for statewide smoke-free policies, and considerable progress has been made in implementing local level smoke-free policies in many states;^§^ therefore, workers classified as being unprotected by statewide laws might have been protected by local level laws. Finally, variable distribution of industries by state might have led to some confounding.

Workplace SHS exposure is harmful for workers’ health. In this study, nonsmoking workers residing in states without comprehensive smoke-free laws and those employed in certain industries were more likely to be frequently exposed to workplace SHS. NIOSH encourages employers, especially those in industries with high prevalences of SHS exposure, to implement workplace-specific smoke-free policies to complement state and local smoke-free laws to help reduce SHS exposure among workers and protect workers’ health (*10*).

**Summary**

**What is already known about this topic?**

Secondhand tobacco smoke (SHS) exposure contributes to diseases including heart disease, lung cancer, and stroke. Implementation of smoke-free laws has reduced SHS exposure.

**What is added by this report?**

Nonsmoking workers residing in states without comprehensive smoke-free laws and workers employed in certain industries were more likely to be frequently exposed to workplace SHS. Industry subcategories with the highest prevalences of SHS exposure, and the industry category with the highest number of exposed workers (construction), include outdoor workplaces and other settings unlikely to be protected by smoke-free laws.

**What are the implications for public health practice?**

Implementation of workplace smoke-free policies can help reduce SHS exposure among workers and protect workers’ health.

## Footnotes

* Employed workers were those who were working for pay at a job or business, had a job or business but were not working (i.e., on leave), or were working without pay at a family-owned business. Respondents who were employed in military-specific industries or occupations or with missing industry/occupation information were excluded from the study population.

† https://www.cdc.gov/statesystem.

§ https://www.cdc.gov/mmwr/volumes/65/wr/mm6524a4.htm.
